# Phenotyping cardiogenic shock: an insight from the gulf cardiogenic shock registry

**DOI:** 10.3389/frai.2026.1744896

**Published:** 2026-03-03

**Authors:** Ahmed Elmahrouk, Amin Daoulah, Ahmed Jamjoom, Nooraldaem Yousif, Wael Almahmeed, Prashanth Panduranga, Abdulrahman Arabi, Omar Kanbr, Hatem M. Aloui, Mohammed Alshehri, Badr Alzahrani, Shaber Seraj, Adnan Hussien, Waleed Alharbi, Mohammed A. Qutub, Mokhtar Kahin, Abdullah Alenezi, Mohamed Ajaz Ghani, Taher Hassan, Rajesh Rajan, Said Al Maashani, Abdulwali Abohasan, Mohammed Balghith, Ziad Dahdouh, Abdulrahman M. Alqahtani, Ibrahim A. M Abdulhabeeb, Mohammed Al Jarallah, Mubarak Abdulhadi Aldossari, Harvey Anthony, Mohammed Awad Ashour Awad Ashour, Tarique Shahzad Chachar, Hassan Khan, Abeer M. Shawky, Youssef Elmahrouk, Amir Lotfi, Amr Arafat

**Affiliations:** 1 King Faisal Specialist Hospital and Research Centre - Jeddah, Jeddah, Saudi Arabia; 2 Faculty of Medicine, Tanta University, Tanta, Egypt; 3 Mohammed bin Khalifa bin Salman Al Khalifa Specialist Cardiac Centre, Awali, Bahrain; 4 Cleveland Clinic Abu Dhabi, Abu Dhabi, United Arab Emirates; 5 National Heart Center, Royal Hospital, Muscat, Oman; 6 Hamad Medical Corporation, Doha, Qatar; 7 Faculty of Medicine, Elrazi University, Khartoum, Sudan; 8 King Saud Medical City, Riyadh, Saudi Arabia; 9 Prince Khaled Bin Sultan Cardiac Center, Khamis Mushait, Saudi Arabia; 10 Prince Sultan Cardiac Center, Riyadh, Saudi Arabia; 11 TH Chan School of Medicine, University of Massachusetts Chan Medical School, Worcester, MA, United States; 12 King’s College Hospital London, Jeddah, Saudi Arabia; 13 Cardiac Sciences Department, King Saud University, Riyadh, Saudi Arabia; 14 Department of Medicine, King Abdulaziz University, Jeddah, Saudi Arabia; 15 International Medical Center, Jeddah, Saudi Arabia; 16 Chest Diseases Hospital, Kuwait City, Kuwait; 17 Department of Cardiology, Madinah Cardiac Center, Almadinah, Saudi Arabia; 18 Department of Cardiology, Bugshan General Hospital, Jeddah, Saudi Arabia; 19 Al-Amiri Hospital, Kuwait City, Kuwait; 20 Salalah Heart Center, Sultan Qaboos Hospital, Salalah, Oman; 21 Central Hospital Hafr Albatin, Hafr Albatin, Saudi Arabia; 22 College of Medicine, King Saud bin Abdulaziz University for Health Sciences College of Public Health and Health Informatics, Riyadh, Saudi Arabia; 23 King Faisal Specialist Hospital and Research Centre, Riyadh, Saudi Arabia; 24 King Salman Heart Center, King Fahad Medical City, Riyadh, Saudi Arabia; 25 King Abdulaziz Specialist Hospit, Aljawf, Saudi Arabia; 26 The Royal Hospital National Heart Center, Muscat, Oman; 27 Faculty of Medicine, Al-Azhar University, Cairo, Egypt; 28 Dr Erfan and Bagedo General Hospital, Jeddah, Saudi Arabia; 29 Department of Cardiovascular Medicine, University of Massachusetts Chan Medical School, Worcester, MA, United States; 30 Research and Innovation Institute, Ministry of Defense Health Services, Riyadh, Saudi Arabia

**Keywords:** cardiogenic shock, cluster analysis, machine learning, multi-organ failure, phenotyping, prognosis

## Abstract

**Background:**

Cardiogenic shock (CS) is a life-threatening condition characterized by clinical heterogeneity and high mortality. A “one-size-fits-all” approach to management may be suboptimal. We aimed to identify distinct clinical phenotypes of CS using an unsupervised machine learning approach and to characterize their associated mortality and SCAI stages.

**Methods:**

We conducted a retrospective analysis of 1,513 patients with CS from the Gulf registry. An unsupervised machine learning methodology was employed, using agglomerative hierarchical clustering on seven key continuous variables (Age, Ejection Fraction, Mean Arterial Pressure, Lactate, pH, Creatinine, and Alanine Transaminase) to identify patient subgroups. The optimal number of clusters was determined using a combination of quantitative metrics and clinical interpretability. The identified phenotypes were then validated against external outcomes, including in-hospital mortality and SCAI Shock Stage.

**Results:**

Four distinct clinical phenotypes were identified. Phenotype 1 (“Compensated Low-Risk,” *n* = 492, 32.5%) had the lowest mortality rate (22.4%). Phenotype 2 (“Metabolic Dysfunction,” *n* = 418, 27.6%) was characterized by severe left ventricular dysfunction and had a mortality of 41.9%. Phenotype 3 (“Multi-organ Failure,” *n* = 204, 13.5%) presented with severe metabolic, renal, and hepatic derangement and had the highest mortality (78.4%). Phenotype 4 (“Elderly Decompensated,” *n* = 399, 26.4%) included older patients with moderate metabolic dysfunction and had a mortality of 60.7%. A steep mortality gradient was observed across the phenotypes (*p* < 0.001), and the distribution of SCAI shock stages differed significantly, aligning with the risk profile of each cluster.

**Conclusion:**

In a large, contemporary registry of CS patients, an unsupervised machine learning approach successfully identified four distinct and prognostically significant phenotypes. These data-driven phenotypes, characterized by unique clinical and biomarker profiles, provide a novel framework for risk stratification that moves beyond traditional classification systems and may facilitate the development of personalized therapeutic strategies for cardiogenic shock.

## Background

Cardiogenic shock (CS) remains a leading cause of mortality in patients hospitalized with acute myocardial infarction (MI), with in-hospital death rates persistently remaining between 40% and 50% despite advances in revascularization and mechanical circulatory support (MCS) (van Diepen et al., 2017; Qutub, 2025; Holger et al., 2025). A primary challenge in managing CS is its profound clinical heterogeneity. Patients present with a broad spectrum of hemodynamic derangements, degrees of end-organ dysfunction, and underlying etiologies, making uniform treatment strategies largely ineffective (Harjola et al., 2015).

The recognition of this heterogeneity has spurred interest in clinical phenotyping, which is the process of identifying distinct, clinically meaningful patient subgroups within a larger disease population (Møller et al., 2025). Such an approach, a cornerstone of precision medicine, aims to move beyond a “one-size-fits-all” paradigm toward tailored therapeutic strategies. In cardiovascular disease, phenotyping has successfully reclassified conditions like heart failure, identifying subgroups with different underlying pathophysiology and responses to treatment (Shah et al., 2014; Kao et al., 2015).

The advent of machine learning (ML), particularly unsupervised clustering algorithms, provides a powerful, data-driven toolkit to uncover these latent clinical phenotypes without preconceived hypotheses (Seymour et al., 2019). By analyzing complex, high-dimensional clinical data, ML can identify patterns and relationships that are not readily apparent to clinicians, thereby defining subgroups based on their intrinsic biological and clinical characteristics (Finlayson et al., 2021).

While previous studies have applied clustering to CS, they have often been limited by the inclusion of specific population not representative of the global CS patients (Zweck et al., 2021). The Society for Cardiovascular Angiography and Interventions (SCAI) classification has provided a crucial framework for staging CS severity, but it primarily reflects a continuum of hemodynamic compromise rather than distinct pathophysiological phenotypes (Naidu et al., 2022; Rajan et al., 2025). The SCAI classification relies primarily on clinical and hemodynamic parameters assessed at discrete time points and may not fully capture the multidimensional heterogeneity inherent in this syndrome (Rajan et al., 2025). Traditional classification systems are limited by their categorical nature, which may oversimplify the complex interplay of metabolic, hemodynamic, and end-organ dysfunction that characterizes cardiogenic shock. Furthermore, these systems were developed based on expert consensus rather than data-driven analysis of patient outcomes. In contrast, unsupervised machine learning approaches can simultaneously integrate multiple clinical variables to identify naturally occurring patient subgroups without *a priori* assumptions, potentially revealing phenotypes that are more biologically and prognostically meaningful. This data-driven methodology may uncover hidden patterns and risk factors that are not apparent through conventional classification, thereby enabling more precise risk stratification and individualized therapeutic strategies (Rauseo et al., 2025).

This study leverages a large, contemporary cohort of CS patients from the Gulf registry. We aimed to identify distinct clinical phenotypes of CS using an unsupervised machine learning approach and to characterize their associated mortality and SCAI stages. We hypothesized that this approach would reveal prognostically significant subgroups with unique clinical and biomarker profiles, offering novel insights into the prognosis of CS and providing a framework for improved risk stratification and personalized care.

## Methods

### Study population and data source

This analysis was conducted on a retrospective cohort of 1,513 patients with cardiogenic shock from the Gulf registry of cardiogenic shock (Daoulah et al., 2024). The dataset encompasses a comprehensive range of variables, including patient demographics, clinical presentation, comorbidities, laboratory findings, hemodynamic parameters, and in-hospital outcomes. All data were collected at the time of initial hospital admission. The study was approved by the local institutional review boards, and the requirement for informed consent was waived due to the retrospective and de-identified nature of the data.

Cardiogenic shock was defined based on established clinical criteria, including sustained hypotension (systolic blood pressure ≤90 mmHg for at least 30 min or the need for vasoactive agents), evidence of end-organ hypoperfusion, and cardiac dysfunction as the primary cause (van Diepen et al., 2017). Details of the Gulf-CS registry were previously published (Daoulah et al., 2024; Arabi et al., 2025; Daoulah et al., 2025).

### Data analysis

**Figure 1:**
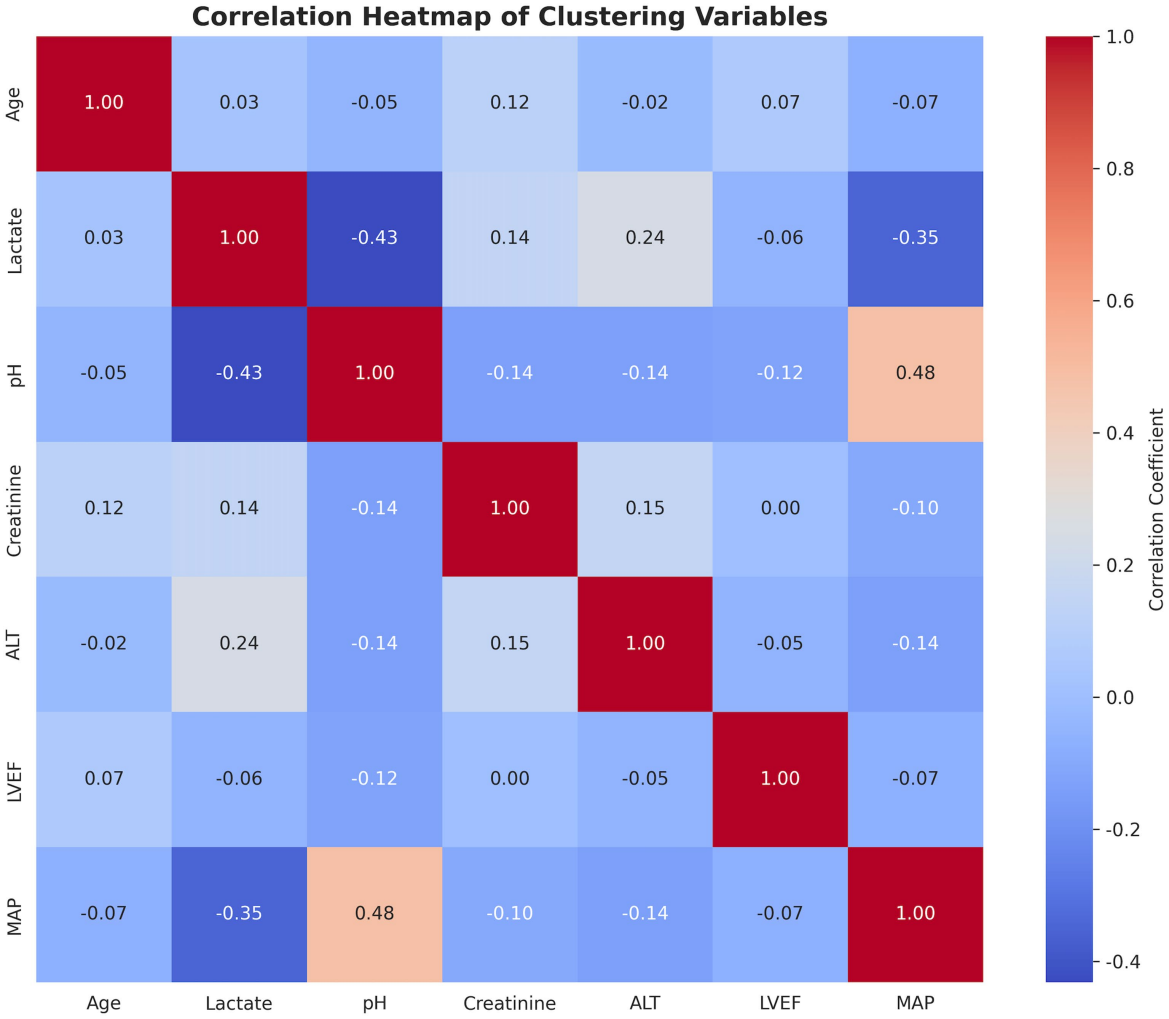
Correlation matrix heatmap of clustering variables. ALT, Alanine Transaminase; LVEF, left ventricular ejection fraction; MAP, mean arterial pressure.

We performed an unsupervised cluster analysis to identify distinct clinical phenotypes among patients in the registry. To ensure the phenotypes were defined by underlying physiology and the clinical relevance, we restricted the clustering algorithm to seven continuous physiological and demographic variables: Age, Left Ventricular Ejection Fraction (LVEF), Mean Arterial Pressure (MAP), Lactate, pH, Creatinine, and Alanine Transaminase (ALT). Spearman correlation detected no significant correlations between clustering variables (*r* < 0.6) (Figure 1).

Variables acting as “binary splitters” (specifically, *In-Hospital Cardiac Arrest*) were excluded from the clustering input to prevent the algorithm from segregating patients solely based on a single binary event.

### Data preprocessing

We first assessed data completeness; variables with >10% missing data were excluded. This resulted in the removal of C-reactive protein (CRP), smoking status, and aspartate aminotransferase (AST). For retained variables, missing values were imputed using the median to minimize the impact of outliers. Higher proportions of missing data can introduce significant bias and reduce the accuracy of imputation methods, particularly in unsupervised learning contexts where the underlying data structure is being discovered rather than predicted (Pereira et al., 2024). The excluded variables did not affect model performance, as they showed no significant association with mortality in a previous analysis (Daoulah et al., 2024). To address the significant right-skewness observed in metabolic markers, we applied a Power Transformation using the Yeo-Johnson method. This transformation stabilizes variance and normalizes distributions, satisfying the assumptions of distance-based clustering algorithms.

### Clustering algorithm

We employed Agglomerative Hierarchical Clustering to define patient subgroups. The dissimilarity between patients was calculated using Gower’s Distance (via the gower Python library). To identify compact and homogeneous clusters, we used Ward’s Minimum Variance Linkage method. A critical methodological adaptation was applied: the Gower distance matrix was squared prior to linkage. This step ensures mathematical consistency with Ward’s algorithm, which minimizes the within-cluster sum of squares and implicitly assumes squared Euclidean distances.

### Cluster determination and validation

**Figure 2:**
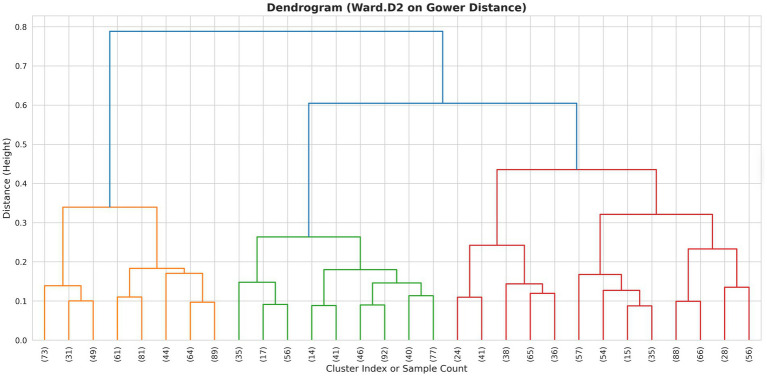
Hierarchical clustering dendrogram. A dendrogram illustrating the results of agglomerative hierarchical clustering using the Ward.D2 linkage method on a Gower distance matrix. The *y*-axis (Height) represents the distance at which clusters are merged, with lower values indicating higher similarity. The *x*-axis corresponds to individual samples or cluster indices.

The optimal number of clusters (k) was determined through a combination of quantitative metrics and clinical interpretability:

1. *Dendrogram analysis:* Visual inspection of the hierarchical tree to identify natural cut points (Figure 2).
2. *Elbow method:* Analysis of the aggregation height (within-cluster variance) plot to detect the inflection point where marginal distinctness decreased.
3. *Silhouette score:* Calculation of the average silhouette width for (*k* = 2) to (*k* = 6) to maximize cluster cohesion and separation.

Based on these metrics and the clinical utility, a 4-cluster solution was selected as the optimal model.

### *Post-hoc* profiling and external validation

Following cluster assignment, we characterized the phenotypes by comparing the medians (continuous variables) and proportions (categorical variables) across the four groups.

To validate the clinical relevance of the identified phenotypes, we assessed their association with external outcomes not included in the clustering algorithm: hospital mortality and SCAI Shock Stage (Society for Cardiovascular Angiography and Interventions classification).

### Statistical analysis

Continuous variables were summarized using median and interquartile range (IQR), and differences across clusters were assessed using the Kruskal-Wallis test. *Post-hoc* pairwise comparisons with adjustment for multiple testing (Dunn’s test) were conducted to identify specific inter-phenotype differences. Categorical variables were summarized as counts and percentages, and differences were evaluated using Chi-squared or Fisher’s exact test, when appropriate. A *p* < 0.05 was considered statistically significant for the primary comparisons. Clustering analyses were performed using Python 3.11 with specialized libraries including scikit-learn for machine learning algorithms, pandas for data manipulation, and matplotlib and seaborn for data visualization. Comparisons between clusters were performed using Stata 18.

## Results

### Cluster characteristics

**Figure 3:**
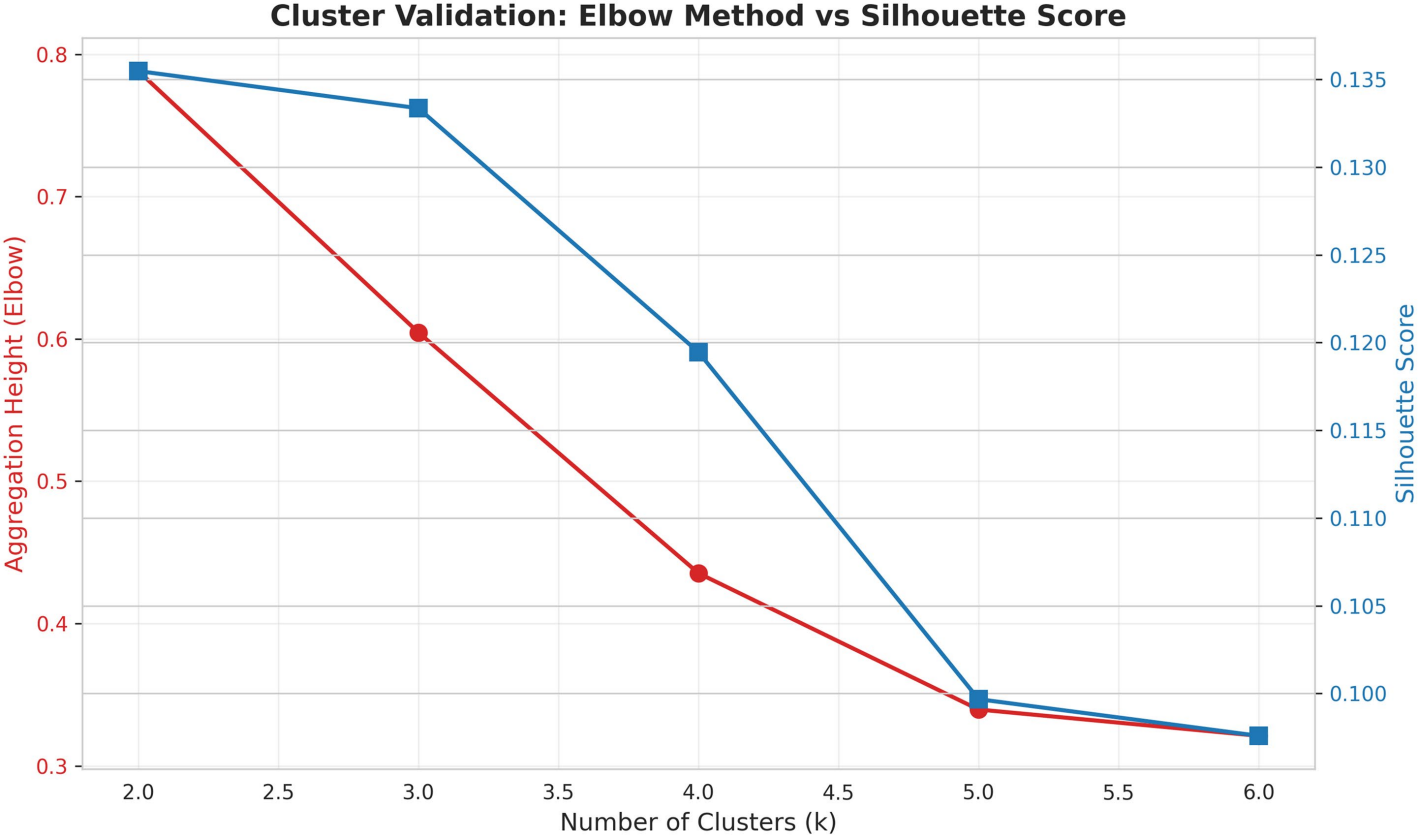
Statistical metrics for determining the optimal number of clusters (*k*). Agglomerative hierarchical clustering was performed. The Elbow method plots the aggregation height (*y*-axis, left) against the number of clusters, *k* (*x*-axis). A sharp decline in aggregation height, indicating a point of diminishing returns. The average silhouette score (*y*-axis, right) measures cluster cohesion and separation, with values approaching 1.0 indicating well-defined clusters.

The analysis of potential cluster numbers for this shock registry dataset yielded several informative metrics. The Silhouette Score at *k* = 4 was 0.1195, indicating only a moderate degree of separation between the resulting clusters. The model’s best internal cohesion and separation, according to this metric, was actually achieved with two clusters, yielding a Silhouette Score of 0.1355. The elbow method aggregation height drops from 0.6046 (*k* = 3) to 0.4354 (*k* = 4), representing a significant jump. Beyond *k* = 4, the improvement level off (Figure 3). A four-cluster model was ultimately selected. This decision was driven by clinical utility and alignment with established standard practice in shock registries, where a four-cluster framework provides a more nuanced and actionable stratification of patient phenotypes for clinical research and application.

- Cluster 1: “Compensated Low-Risk Phenotype” (*n* = 492, 32.5% of patients)
- Cluster 2: “Metabolic Dysfunction Phenotype” (*n* = 418, 27.6% of patients)
- Cluster 3: “Multi-organ Failure Phenotype” (*n* = 204, 13.52% of patients)
- Cluster 4: “Elderly Decompensated Phenotype” (*n* = 399, 26.4% of patients)

*Phenotype 1* represents the lowest risk phenotype with the best compensatory mechanisms. There is minimal metabolic acidosis and lactate elevation, and the best preserved perfusion pressure and acid-base balance.

*Phenotype 2* is characterized by severe left ventricular dysfunction with a low ejection fraction (the lowest LVEF; median = 21%). The patients have significant hepatic involvement with elevated ALT. Lactate is moderately elevated.

*Phenotype 3* represents patients with multi-organ failure with severe metabolic derangement. The patients suffer from severe renal dysfunction with the highest creatinine, severe hepatic injury (highest ALT; median = 300 U/L), and severe lactic acidosis.

*Phenotype 4* include elder patients with moderate metabolic derangement and moderate elevation of all markers, with balanced presentation of cardiogenic and metabolic dysfunction.

Table 1 presents the distribution of clustering variables across the derived phenotypes. Compariosns revealed statistically significant differences across phenotypes for all evaluated variables: age (*p* < 0.001), pH (*p* < 0.001), mean arterial pressure (MAP, *p* < 0.001), left ventricular ejection fraction (LVEF, *p* < 0.001), creatinine (*p* < 0.001), and alanine aminotransferase (ALT, *p* < 0.001). *Post-hoc* pairwise comparisons with adjustment for multiple testing were conducted to identify specific inter-phenotype differences. This analysis showed that age did not differ significantly between Phenotypes 1 and 3 (adjusted *p*=0.093). Similarly, no significant pairwise difference was found for pH between Phenotypes 3 and 4 (adjusted *p* = 0.336), for MAP between Phenotypes 3 and 4 (adjusted *p* = 0.135), or for LVEF between Phenotypes 1 and 3 (adjusted *p* = 0.066) and Phenotypes 3 and 4 (adjusted *p* = 0.328). For creatinine, the pairwise comparison between Phenotypes 2 and 4 was not statistically significant (adjusted *p* = 0.054). In contrast, ALT demonstrated statistically significant pairwise differences between all phenotypes (all adjusted *p* < 0.001).

**Table 1:** Comparison of the clustering variables among the three phenotypes.

Clustering variables	Phenotype 1 (*n* = 492)	Phenotype 2 (*n* = 418)	Phenotype 3 (*n* = 204)	Phenotype 4 (*n* = 399)	*p*-value
Age, years	58 (51–67)	56 (50–65)	60 (55–65.5)	68 (57–76)	<0.001
pH	7.35 (7.3–7.41)	7.29 (7.21–7.35)	7.12 (7.04–7.27)	7.19 (7.1–7.27)	<0.001
Lactate (mmol/L)	1.2 (1–1.8)	2.6 (1.8–4)	3.8 (2.4–7.4)	3.2 (2.1–4.9)	<0.001
LVEF (%)	35 (27–42)	21 (17–25)	36 (30–42)	36 (31–42)	<0.001
MAP (mmHg)	55 (53–57)	53 (50–56)	50 (45–55)	50 (45–53)	<0.001
Creatinine (μmol/L)	91 (70–115)	108 (86–138)	180 (113–220)	110 (86–166)	<0.001
ALT (U/L)	30 (22–50)	68 (40–131)	300 (197–670)	45 (26–65)	<0.001

Data are presented as median (25th–75th percentiles) or numbers (%). ALT, Alanine Aminotransferase; MAP, Mean Arterial Pressure; LVEF, left ventricular ejection fraction.

### Clusters comparison

The baseline clinical, presentation, and angiographic characteristics of the patients stratified into the four phenotypes are presented in Table 2. Significant differences were observed across multiple variables.

**Table 2:** Comparison of the baseline characteristics, presentation, and cardiac function of the clusters.

Variables	Phenotype 1 (*n* = 492)	Phenotype 2 (*n* = 418)	Phenotype 3 (*n* = 204)	Phenotype 4 (*n* = 399)	*p*-value
Diabetes mellitus	298 (60.57%)	263 (62.92%)	134 (65.69%)	262 (65.66%)	0.382
Previous MI	139 (28.25%)	67 (16.03%)	41 (20.10%)	96 (24.06%)	<0.001
History of CABG	21 (4.27%)	13 (3.11%)	7 (3.34%)	14 (3.51%)	0.817
Peripheral arterial disease	25 (5.08%)	15 (3.59%)	16 (7.84%)	20 (5.01%)	0.157
Cerebrovascular accident	32 (6.50%)	17 (4.07%)	15 (7.35%)	43 (10.78%)	0.002
Congestive heart failure	63 (12.80%)	44 (10.53%)	20 (9.80%)	45 (11.28%)	0.615
STEMI	344 (69.92%)	343 (82.06%)	160 (78.43%)	270 (67.67%)	<0.001
In-hospital cardiac arrest	142 (28.86%)	213 (50.96%)	140 (68.63%)	209 (52.38%)	<0.001
RV dysfunction	60 (12.32%)	73 (17.98%)	55 (26.96%)	59 (14.94%)	<0.001
Troponin (ng/L)	5.5 (0.64–86)	14.5 (0.73–385)	25 (5–2334)	90 (3–720)	<0.001
SYNTAX	25 (18–32)	24 (19–30)	26 (18–32)	27 (21–33)	<0.001

Data are presented as numbers (%) or median (25th–75th percentiles). CABG, Coronary Artery Bypass Grafting; MI, Myocardial Infarction; RV, Right Ventricle; STEMI, ST-Elevation Myocardial Infarction.

Regarding medical history, the prevalence of diabetes mellitus was high and comparable across all four phenotypes (*p* = 0.382). However, significant inter-phenotype differences were noted in prior cardiovascular events. A history of previous myocardial infarction (MI) was most prevalent in Phenotype 1 (28.25%) and significantly less common in the other groups (*p* < 0.001). Similarly, the incidence of prior cerebrovascular accident varied significantly; Phenotype 4 had the highest CVA prevalence (10.78%, *p* = 0.002). No significant differences were found in the history of CABG, peripheral arterial disease, or congestive heart failure (*p* > 0.05 for all).

Marked disparities were evident in the clinical presentation. Phenotype 2 and Phenotype 3 had the highest proportions of patients presenting with ST-elevation myocardial infarction (STEMI) (82.06 and 78.43%, respectively). In-hospital cardiac arrest was a frequent complication, with its incidence escalating from 28.86% in Phenotype 1 to a peak of 68.63% in Phenotype 3 (*p* < 0.001). The prevalence of right ventricular (RV) dysfunction also differed significantly, being highest in Phenotype 3 (26.96%) and lowest in Phenotype 1 (12.32%, *p* < 0.001).

Biochemical and angiographic severity further distinguished the groups. Peak troponin levels increased progressively across the phenotypes, with median values rising from 5.5 ng/L in Phenotype 1–90 ng/L in Phenotype 4 (*p* < 0.001). There was no difference in troponin between Phenotype 3 and (*p* = 0.386). The angiographic complexity of coronary artery disease, as quantified by the SYNTAX score, was significantly different, with median scores ranging from 24 in Phenotype 2–27 in Phenotype 4 (*p* < 0.001). Phenotype 4 had the highest SYNTX score with a significant difference between Phenotype 1 (*p* < 0.001), Phenotype 2 (*p* < 0.001), and Phenotype 3 (*p* = 0.017).

### Outcomes and SCAI stage

**Figure 4:**
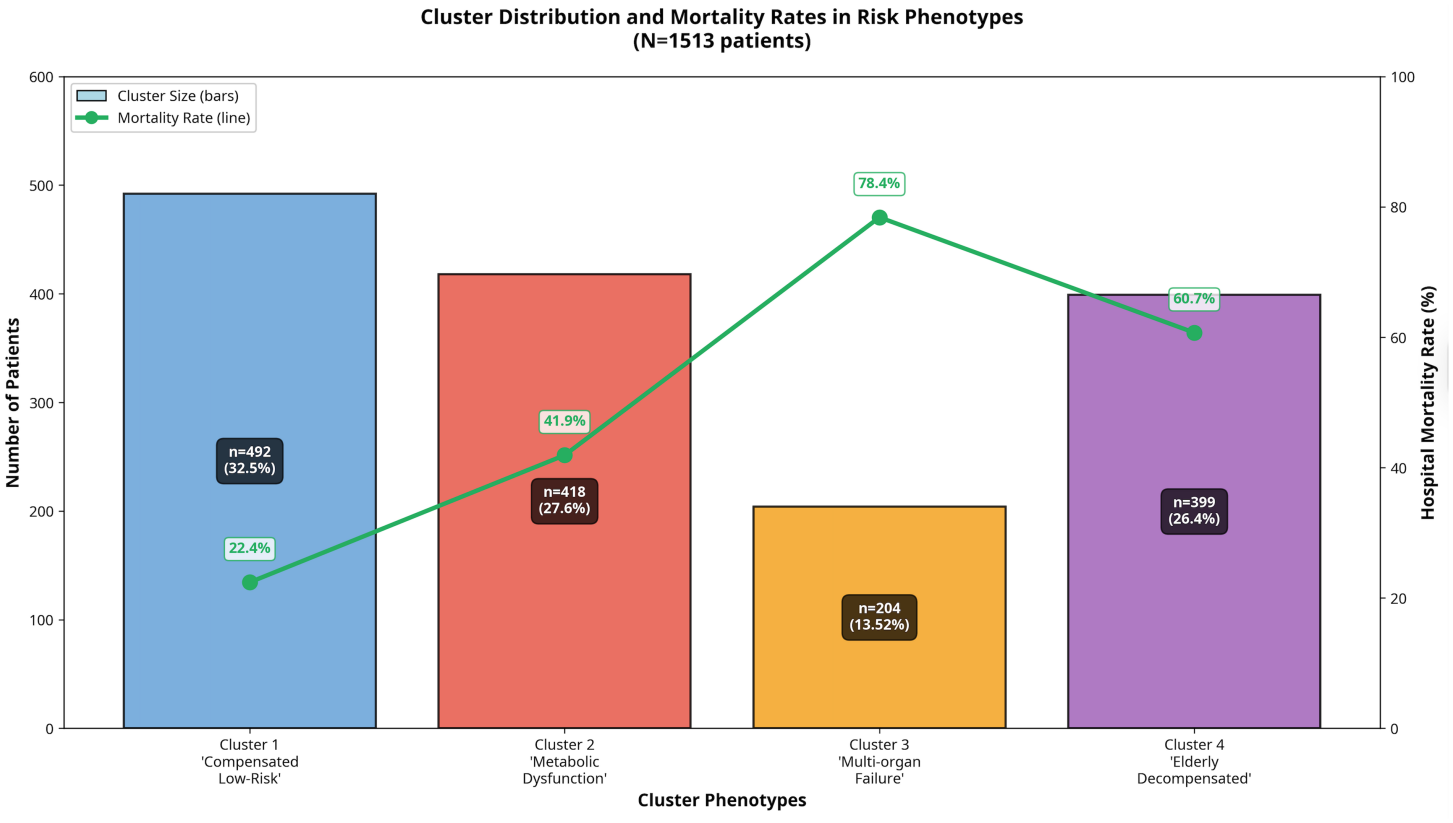
Mortality rates and patient distribution across phenotypes. This combination chart provides a clear overview of the patient distribution and associated mortality for the four identified cardiogenic shock phenotypes. The bar chart (primary *y*-axis) displays the total number of patients within each cluster. The line plot (secondary *y*-axis) illustrates the differences in in-hospital mortality rates corresponding to each phenotype.

A marked gradient in mortality risk was observed across the four phenotypically distinct clusters. Phenotype 1 demonstrated the lowest mortality rate (22.4%) and was therefore designated the reference or low-risk group. In comparison, mortality escalated progressively across the remaining clusters, with rates of 41.9% in Phenotype 2, 60.7% in Phenotype 4, and 78.4% in Phenotype 3. Relative to Phenotype 1, this represented a progressively increasing hazard, with mortality risk approximately 2.0-fold higher in Phenotype 2, 2.7-fold higher in Phenotype 4, and 3.5-fold higher in Phenotype 3 (Figure 4).

**Figure 5:**
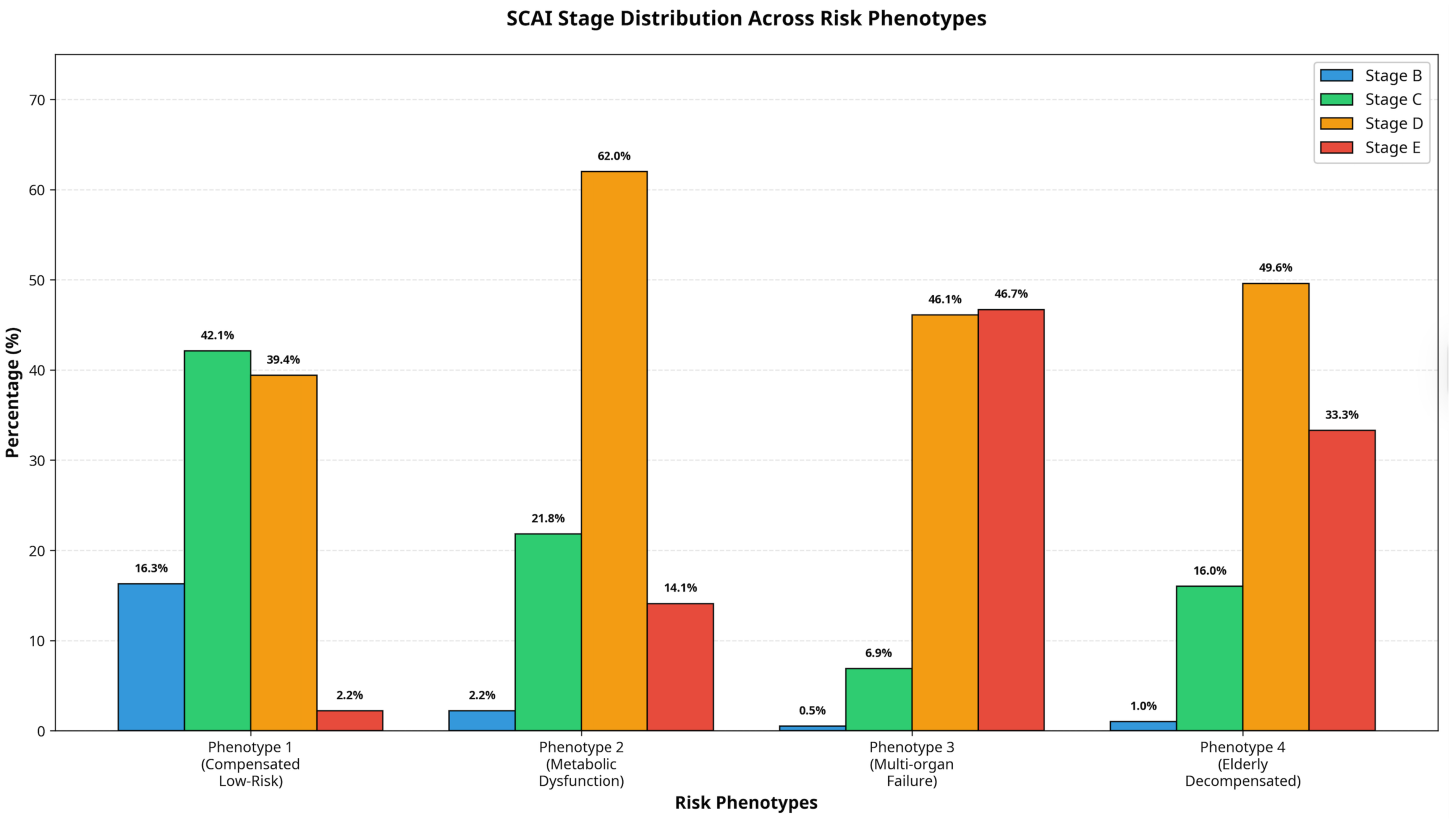
SCAI stage distribution across cardiogenic shock phenotypes.

The distribution of SCAI shock stages differed significantly across the four phenotypes (*p* < 0.001). Phenotype 1 was characterized by a broad distribution across stages B (16.3%), C (42.1%), and D (39.4%), with minimal representation in stage E (2.2%). Phenotype 2 was predominantly comprised of patients in SCAI stages D (62.0%) and C (21.8%), with notable proportions in stages E (14.1%) and B (2.2%). In stark contrast, Phenotype 3 was heavily skewed toward the most severe stages, with near-equal proportions in stages E (46.7%) and D (46.1%), and minimal representation in stages C (6.9%) and B (0.5%). Phenotype 4 demonstrated an intermediate-to-high-risk profile, with the majority of patients in stages D (49.6%) and E (33.3%) (Figure 5).

## Discussion

In this large, contemporary registry of patients with cardiogenic shock, we utilized an unsupervised machine learning approach to identify four distinct clinical phenotypes with markedly different prognostic implications. Our analysis moves beyond traditional, single-parameter risk scores and the established SCAI classification system, providing a data-driven framework that integrates multiple physiological domains to reveal the underlying heterogeneity of CS. The four identified phenotypes, “Compensated Low-Risk,” “Metabolic Dysfunction,” “Multi-organ Failure,” and “Elderly Decompensated,” demonstrate unique clinical, biochemical, and outcome profiles, highlighting the potential for this classification to guide personalized therapeutic strategies.

Phenotype 1, the “Compensated Low-Risk” group, represented the largest and most stable cohort, characterized by preserved end-organ function, minimal metabolic derangement, and the lowest mortality rate (22.4%). These patients align with the traditional presentation of early-stage CS, where compensatory mechanisms are still largely intact (Thiele et al., 2019). Their favorable outcome underscores the importance of early recognition and intervention before the cascade of multi-organ dysfunction ensues (van Diepen et al., 2017; Baran et al., 2019). This group had a higher prevalence of prior MI, suggesting that pre-existing cardiac remodeling might influence the initial response to an acute insult (Jentzer, 2020; Jentzer et al., 2019).

Phenotype 2, the “Metabolic Dysfunction” group, was distinguished by profound left ventricular systolic dysfunction (lowest LVEF) and significant hepatic injury, with a mortality rate of 41.9%. This phenotype likely represents a state of primary cardiac failure where the initial hemodynamic insult rapidly translates into end-organ hypoperfusion, particularly affecting the liver. The high prevalence of STEMI in this group (82.1%) supports the notion of an acute, severe ischemic event driving this presentation (Hochman et al., 1999). This phenotype highlights the critical interplay between cardiac function and hepatic perfusion, where liver injury is not just a marker of passive congestion but an active contributor to the shock state through impaired lactate clearance and systemic inflammation (Lassus, 2020; Biancari Fausto et al., 2023).

Phenotype 3, the “Multi-organ Failure” group, constituted the smallest but most critically ill cohort, with the highest mortality rate at an alarming 78.4%. These patients exhibited a catastrophic failure of multiple organ systems, with severe renal and hepatic dysfunction, profound lactic acidosis, and the highest incidence of in-hospital cardiac arrest. This phenotype represents the terminal stage of the shock spiral, where systemic inflammation, metabolic collapse, and hemodynamic failure are deeply intertwined and mutually reinforcing (van Diepen et al., 2017; Lassus, 2020). The extremely high mortality in this group, despite aggressive care (as evidenced by the high proportion in SCAI Stage E), suggests that by the time patients reach this stage, conventional therapies may be futile (Baran et al., 2019). This finding strongly supports earlier, more aggressive interventions in patients who show signs of progression toward this phenotype.

Phenotype 4, the “Elderly Decompensated” group, comprised older patients with a balanced presentation of moderate cardiac and metabolic dysfunction, yet a very high mortality rate of 60.7%. These patients had the highest SYNTAX scores, indicating more complex coronary artery disease, and the highest prevalence of prior cerebrovascular accidents, reflecting a greater burden of systemic atherosclerosis and reduced physiological reserve (Mack et al., 2013). Their high mortality, despite having less severe individual organ derangements than Phenotype 3, highlights the profound impact of age and comorbidities on CS outcomes. This phenotype underscores the concept of “homeostenosis,” where the aging process diminishes the body’s ability to withstand physiological stress, leading to rapid decompensation and poor outcomes even with seemingly moderate insults (Ferrucci and Fabbri, 2018; Clegg et al., 2013).

Our work builds upon and extends previous efforts to phenotype CS using machine learning techniques. A landmark study by Zweck et al. identified three distinct phenotypes in a cohort of CS patients: a “non-congested” phenotype with the lowest mortality, a “cardiorenal” phenotype with intermediate mortality, and a “cardiometabolic” phenotype with the highest mortality (Zweck et al., 2021). The Zweck et al. study emphasized the roles of congestion, renal dysfunction, and metabolic parameters, whereas our analysis identified multi-organ failure as the primary driver of the high-risk phenotype. This difference may be attributable to the distinct patient populations and the specific variables included in the clustering analysis. Our study, drawing from the Gulf-CS registry, includes a younger patient population with a higher prevalence of diabetes, which may contribute to different pathophysiological manifestations of CS (Daoulah et al., 2024). Despite these differences, both studies converge on the central theme that CS is not a monolithic entity, and that data-driven phenotyping can reveal prognostically relevant subgroups. More recently, Jentzer et al. have reviewed machine learning approaches for phenotyping in cardiogenic shock and critical illness, emphasizing the potential of unsupervised clustering methods such as k-means, hierarchical clustering, and latent class analysis to identify subphenotypes that may respond differently to treatment (Jentzer et al., 2022a).

This work aligns with a growing body of literature emphasizing that CS is not merely a state of cardiac pump failure but a systemic disease culminating in multi-organ dysfunction syndrome (MODS) (Lassus, 2020; Shirakabe et al., 2023). Recent findings have confirmed that noncardiac organ failure is prevalent and rising in patients with acute myocardial infarction-related CS, and its presence is a powerful predictor of a grim prognosis, often trumping the success of revascularization (Vallabhajosyula et al., 2019). The metabolic derangements observed in our Phenotype 2 are consistent with the concept of “metabolic shock” (Jentzer et al., 2022b). Elevated lactate, a marker of tissue hypoperfusion and anaerobic metabolism, has been consistently associated with increased mortality in CS (Pagnesi et al., 2025). Moreover, the rate of lactate clearance has emerged as a powerful prognostic indicator, with slower clearance portending a worse prognosis (Marbach et al., 2022). Our findings reinforce the importance of early and serial lactate measurements in risk-stratifying patients with CS. The significant metabolic acidosis in our higher-risk clusters further underscores the severity of circulatory failure and its systemic consequences. Jentzer et al. have shown that higher lactate and lower pH predict mortality in patients with CS beyond standard measures of shock severity, suggesting that these metabolic markers capture a dimension of illness severity that is not fully reflected in hemodynamic parameters alone (Jentzer et al., 2022c). These metabolic derangements, coupled with evidence of renal and hepatic dysfunction, paint a picture of a downward spiral of multi-organ failure that is particularly pronounced in the multi-organ failure phenotype.

The correlation between our clusters and the SCAI stages of CS further validates our findings: the low-risk cluster predominantly comprises patients in SCAI stages B and C, and the multi-organ failure cluster is dominated by stage E. This alignment with an established risk stratification scheme strengthens the clinical relevance of our data-driven phenotypes.

Our study has several important clinical implications. First, the identification of these distinct phenotypes can help clinicians to more accurately risk-stratify patients with CS at the bedside, moving beyond a “one-size-fits-all” approach. The simple, readily available clinical and laboratory variables used to define our clusters, including pH, creatinine, lactate, MAP, LVEF, and ALT, can be easily integrated into clinical practice. Second, this phenotyping framework can guide resource allocation and the selection of appropriate therapeutic strategies. For example, patients in the low-risk phenotype may be managed with a more conservative approach, avoiding the potential complications of unnecessary invasive procedures. Conversely, patients identified as metabolic shock may benefit from earlier and more aggressive interventions, such as the early initiation of mechanical circulatory support. Third, our findings can inform the design of future clinical trials in CS. By enrolling more homogeneous patient populations based on their clinical phenotype, future trials may be better powered to detect the efficacy of novel therapies. This approach is consistent with the broader movement toward precision medicine in cardiovascular disease, which seeks to tailor treatment to the individual characteristics of each patient (Zweck et al., 2025).

### Limitations

This study has several important limitations that must be acknowledged. First, its retrospective design, based on a registry, is susceptible to selection bias and unmeasured confounding, although the Gulf-CS registry is one of the largest and most detailed contemporary CS databases. Second, the analysis was based on data collected at the time of initial hospital admission, providing a static snapshot of a dynamic and evolving syndrome. We did not capture the temporal evolution of shock or the impact of specific interventions on patient trajectories. Future studies should incorporate serial measurements to model the dynamic nature of CS.

Third, the process of unsupervised clustering involves several methodological choices, including the selection of input variables and the determination of the optimal number of clusters. While we used a data-driven approach to select mortality-relevant variables and a combination of statistical metrics and clinical utility to select the four-cluster solution, this choice is inherently subjective. The Silhouette Score suggested that a two-cluster solution would have better internal cohesion, but a four-cluster model was chosen for its superior clinical interpretability and actionability, a common practice in medical phenotyping research. Fourth, data on certain potentially important variables, such as inflammatory biomarkers (e.g., CRP) and right heart catheterization parameters, were incomplete, precluding their inclusion in the clustering model. The exclusion of these variables might have limited the granularity of the resulting phenotypes. Finally, while our findings were internally validated by their strong association with mortality and SCAI stage, they require external validation in an independent cohort to confirm their generalizability and transportability to other patient populations. Our cohort is from the Gulf region; its demographic and etiological profile (e.g., high prevalence of diabetes, younger age at presentation) may differ from North American or European cohorts. Therefore, the generalizability of these specific phenotypes needs to be validated in external datasets.

## Conclusion

This study successfully applied an unsupervised machine learning algorithm to a large, contemporary cohort of cardiogenic shock patients, identifying four distinct and prognostically significant clinical phenotypes. These data-driven phenotypes, defined by unique combinations of demographic, hemodynamic, and metabolic characteristics, provide a more nuanced and granular framework for risk stratification than traditional classification systems. This approach moves beyond a “one-size-fits-all” understanding of cardiogenic shock and could offer a foundation for developing phenotype-specific therapeutic strategies. Prospective validation of these phenotypes is warranted to confirm their clinical utility in guiding personalized care and improving the dismal outcomes of this devastating syndrome.

## Data Availability

The raw data supporting the conclusions of this article will be made available by the corresponding author after institutional approvals.

## References

[ref1] Arabi A. Al Suwaidi J. Daoulah A. AlQahtani A. A. Shahid Z. Jamjoom A. . (2025). Timing of mechanical ventilation and its association with in-hospital outcomes in patients with cardiogenic shock following ST-elevation myocardial infarction: a multicentre observational study. BMJ Open 15. doi: 10.1136/bmjopen-2025-099208, 40467313 PMC12142138

[ref2] Baran D. A. Grines C. L. Bailey S. Burkhoff D. Hall S. A. Henry T. D. . (2019). SCAI clinical expert consensus statement on the classification of cardiogenic shock. Catheter. Cardiovasc. Interv. 94, 29–37. doi: 10.1002/ccd.28329, 31104355

[ref3] Clegg A. Young J. Iliffe S. Rikkert M. O. Rockwood K. (2013). Frailty in elderly people. Lancet 381, 752–762. doi: 10.1016/s0140-6736(12)62167-9, 23395245 PMC4098658

[ref4] Daoulah A. Alshehri M. Panduranga P. Aloui H. M. Yousif N. Arabi A. . (2024). Clinical outcomes of patients with cardiogenic shock complicating acute myocardial infarction: the Gulf-Cardiogenic Shock (G-CS) registry. Shock:62, 512–521. doi: 10.1097/SHK.0000000000002433 39158570

[ref5] Daoulah A. Kanbr O. Elmahrouk A. Jarallah Al M. Yousif N. Jamjoom A. . (2025). Clinical characteristics, management, and outcomes of acute myocardial infarction-related cardiogenic shock patients with and without out-of-hospital cardiac arrest: a Gulf region registry analysis. Resusc Plus. 26:101091. doi: 10.1016/j.resplu.2025.101091 41050166 PMC12489831

[ref6] Fausto B. Alexander K. Andrea P. Ruggieri Vito G. Sung-Min C. Kook K. J. . (2023). Hyperlactatemia and poor outcome after postcardiotomy veno-arterial extracorporeal membrane oxygenation: an individual patient data meta-analysis. Perfusion 39, 956–965. doi: 10.1177/02676591231170978 37066850

[ref7] Ferrucci L. Fabbri E. (2018). Inflammageing: chronic inflammation in ageing, cardiovascular disease, and frailty. Nat. Rev. Cardiol. 15, 505–522. doi: 10.1038/s41569-018-0064-2, 30065258 PMC6146930

[ref8] Finlayson S. G. Subbaswamy A. Singh K. Bowers J. Kupke A. Zittrain J. . (2021). The clinician and dataset shift in artificial intelligence. N. Engl. J. Med. 385, 283–286. doi: 10.1056/NEJMc2104626 34260843 PMC8665481

[ref9] Harjola V. P. Lassus J. Sionis A. Køber L. Tarvasmäki T. Spinar J. . (2015). Clinical picture and risk prediction of short-term mortality in cardiogenic shock. Eur J Heart Fail. 17, 501–509. doi: 10.1002/ejhf.260 25820680

[ref10] Hochman J. S. Sleeper L. A. Webb J. G. Sanborn T. A. White H. D. Talley J. D. . (1999). Early revascularization in acute myocardial infarction complicated by cardiogenic shock. SHOCK investigators. Should we emergently revascularize occluded coronaries for cardiogenic shock. N. Engl. J. Med. 341, 625–634. 10460813 10.1056/NEJM199908263410901

[ref11] Holger T. Uwe Z. Franz-Josef N. Miroslaw F. Hans-Georg O. Jörg H. . (2025). Intraaortic balloon support for myocardial infarction with cardiogenic shock. N. Engl. J. Med. 367, 1287–1296. doi: 10.5694/mja2.70127 22920912

[ref12] Jentzer J. C. (2020). Understanding cardiogenic shock severity and mortality risk assessment. Circ. Heart Fail.. United States 13:e007568. doi: 10.1161/CIRCHEARTFAILURE.120.007568 32900232

[ref13] Jentzer J. C. Kashani K. B. Wiley B. M. Patel P. C. Baran D. A. Barsness G. W. . (2022b). Laboratory markers of acidosis and mortality in cardiogenic shock: developing a definition of hemometabolic shock. Shock 57. doi: 10.1097/SHK.0000000000001812 33988540

[ref14] Jentzer J. C. Rayfield C. Soussi S. Berg D. D. Kennedy J. N. . (2022a). Machine learning approaches for phenotyping in cardiogenic shock and critical illness. JACC Adv. 1:100126. doi: 10.1016/j.jacadv.2022.100126 38939698 PMC11198618

[ref15] Jentzer J. C. Schrage B. Patel P. C. Kashani K. B. Barsness G. W. Holmes D. R. . (2022c). Association between the acidemia, lactic acidosis, and shock severity with outcomes in patients with cardiogenic shock. J. Am. Heart Assoc. 11:e024932. doi: 10.1161/JAHA.121.024932, 35491996 PMC9238598

[ref16] Jentzer J. C. van Diepen S. Barsness G. W. Henry T. D. Menon V. Rihal C. S. . (2019). Cardiogenic shock classification to predict mortality in the cardiac intensive care unit. J. Am. Coll. Cardiol. 74, 2117–2128. doi: 10.1016/j.jacc.2019.07.077 31548097

[ref17] Kao D. P. Lewsey J. D. Anand I. S. Massie B. M. Zile M. R. Carson P. E. . (2015). Characterization of subgroups of heart failure patients with preserved ejection fraction with possible implications for prognosis and treatment response. Eur J Heart Fail. 17, 925–935. doi: 10.1002/ejhf.327 26250359 PMC4654630

[ref18] Lassus J. (2020). Kidney and liver dysfunction in cardiogenic shock. Curr. Opin. Crit. Care. 26. doi: 10.1097/MCC.0000000000000746 32520812

[ref19] Mack M. J. Head S. J. Holmes D. R. J. Ståhle E. Feldman T. E. Colombo A. . (2013). Analysis of stroke occurring in the SYNTAX trial comparing coronary artery bypass surgery and percutaneous coronary intervention in the treatment of complex coronary artery disease. JACC Cardiovasc. Interv. 6, 344–354. doi: 10.1016/j.jcin.2012.11.010 23523456

[ref20] Marbach J. A. Di Santo P. Kapur N. K. Thayer K. L. Simard T. Jung R. G. . (2022). Lactate clearance as a surrogate for mortality in cardiogenic shock: insights from the DOREMI trial. J. Am. Heart Assoc. 11:e023322. doi: 10.1161/JAHA.121.023322, 35261289 PMC9075306

[ref21] Møller J. E. Hassager C. Proudfoot A. De Backer D. Morrow D. A. Ravn H. B. . (2025). Cardiogenic shock: diagnosis, phenotyping and management. Intensive Care Med. 51, 1651–1663. doi: 10.1007/s00134-025-08049-y, 40768067

[ref22] Naidu S. S. Baran D. A. Jentzer J. C. Hollenberg S. M. van Diepen S. Basir M. B. . (2022). SCAI SHOCK stage classification expert consensus update: a review and incorporation of validation studies: This statement was endorsed by the American College of Cardiology (ACC), American College of Emergency Physicians (ACEP), American Heart Associatio. J. Am. Coll. Cardiol. 79, 933–946. doi: 10.1016/j.jacc.2022.01.018 35115207

[ref23] Pagnesi M. Riccardi M. Sacco A. Viola G. Oliva F. Frea S. . (2025). Lactate values and mortality in patients with cardiogenic shock: insights from the Altshock-2 registry. Crit. Care Med. 53:2412–2420. doi: 10.1097/ccm.0000000000006738, 40504883

[ref24] Pereira R. C. Abreu P. H. Rodrigues P. P. (2024). Siamese autoencoder architecture for the imputation of data missing not at random. J. Comput. Sci. 78:102269. doi: 10.1016/j.jocs.2024.102269

[ref25] Qutub M. A. (2025). The use of an intra-aortic balloon pump in patients with cardiogenic shock secondary to acute myocardial infarction. Cardiothorac. Surg. 33:7. doi: 10.1186/s43057-025-00151-7

[ref26] Rajan R. Al Jarallah M. Daoulah A. Panduranga P. Elmahrouk A. Mohamed Al Rawahi A. S. . (2025). The utility and validation of SCAI-CSWG stages in patients with acute myocardial infarction-related cardiogenic shock. J. Soc. Cardiovasc. Angiogr. Interv. 4:102461. doi: 10.1016/j.jscai.2024.102461, 40061415 PMC11887555

[ref27] Rauseo E Salih AM Cooper J Abdulkareem M Banerji CRS Chadalavada S et al Clinical phenotypes in hypertension: a data-driven approach to risk stratification. Hypertension, (Epub ahead of print). (2025); doi: 10.1161/HYPERTENSIONAHA.125.25187 PMC13189382 41376588

[ref28] Seymour C. W. Kennedy J. N. Wang S. Chang C. C. H. Elliott C. F. Xu Z. . (2019). Derivation, validation, and potential treatment implications of novel clinical phenotypes for sepsis. JAMA 321, 2003–2017. doi: 10.1001/jama.2019.5791 31104070 PMC6537818

[ref29] Shah S. J. Katz D. H. Deo R. C. (2014). Phenotypic spectrum of heart failure with preserved ejection fraction. Heart Fail Clin. 10, 407–418. doi: 10.1016/j.hfc.2014.04.008 24975905 PMC4076705

[ref30] Shirakabe A. Matsushita M. Shibata Y. Shighihara S. Nishigoori S. Sawatani T. . (2023). Organ dysfunction, injury, and failure in cardiogenic shock. J. Intensive Care 11:26. doi: 10.1186/s40560-023-00676-1, 37386552 PMC10308671

[ref31] Thiele H. Ohman E. M. de Waha-Thiele S. Zeymer U. Desch S. (2019). Management of cardiogenic shock complicating myocardial infarction: an update 2019. Eur Heart J. 40, 2671–2683. doi: 10.1093/eurheartj/ehz363 31274157

[ref32] Vallabhajosyula S. Dunlay S. M. Prasad A. Kashani K. Sakhuja A. Gersh B. J. . (2019). Acute noncardiac organ failure in acute myocardial infarction with cardiogenic shock. J. Am. Coll. Cardiol. 73, 1781–1791. doi: 10.1016/j.jacc.2019.01.053 30975295

[ref33] van Diepen S. Katz J. N. Albert N. M. Henry T. D. Jacobs A. K. Kapur N. K. . (2017). Contemporary management of cardiogenic shock: a scientific statement from the American heart association. Circulation. 136, e232–e268. doi: 10.1161/CIR.0000000000000525 28923988

[ref34] Zweck E. Li S. Burkhoff D. Kapur N. K. (2025). Profiling of cardiogenic shock: incorporating machine learning into bedside management. J. Soc. Cardiovasc. Angiogr. Interv. 4. doi: 10.1016/j.jscai.2024.102047, 40230675 PMC11993856

[ref35] Zweck E. Thayer K. L. Helgestad O. K. L. Kanwar M. Ayouty M. Garan A. R. . (2021). Phenotyping cardiogenic shock. J Am Heart Assoc. 10:e020085. doi: 10.1161/JAHA.120.020085 34227396 PMC8483502

